# (4-Nitro­phen­yl)methanol

**DOI:** 10.1107/S1600536812024865

**Published:** 2012-06-13

**Authors:** Ivana Císařová, Petr Štěpnička

**Affiliations:** a Department of Inorganic Chemistry, Faculty of Science, Charles University in Prague, Hlavova 2030, 12840 Prague 2, Czech Republic

## Abstract

In the crystal of the title compound, C_7_H_7_NO_3_, mol­ecules associate into infinite chains *via* O—H⋯O(NO_2_) hydrogen bonds propagating in the [101] direction. These chains are linked *via* C—H⋯O(NO_2_) hydrogen bonds to form double-stranded ribbons lying parallel to the *ac* plane. The ribbons stack along the *b* axis by means of π–π inter­actions involving the benzene rings and the nitro group. The centroid–centroid distances of the alternating parallel aromatic rings are 3.6514 (7) and 3.8044 (7) Å.

## Related literature

For the crystal structure of a Zn^II^ complex with *O*-coordinated 4-nitro­benzyl alcohol, see: Koller *et al.* (2009 ▶). For a survey of typical bond lengths in organic compounds, see: Allen *et al.* (2006 ▶).
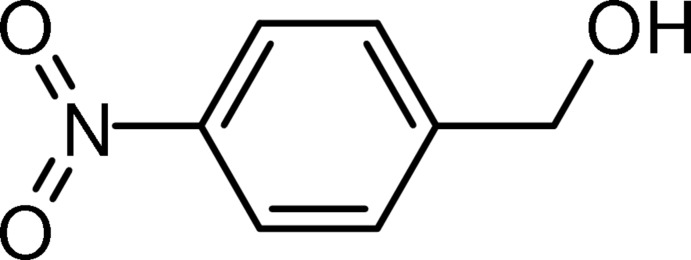


## Experimental

### 

#### Crystal data

C_7_H_7_NO_3_
*M* *_r_* = 153.14
Triclinic, 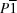

*a* = 6.2216 (5) Å
*b* = 7.4096 (6) Å
*c* = 7.7833 (6) Å
α = 110.867 (2)°
β = 93.667 (2)°
γ = 90.748 (3)°
*V* = 334.34 (5) Å^3^
*Z* = 2
Mo *K*α radiation
μ = 0.12 mm^−1^
*T* = 150 K
0.53 × 0.31 × 0.28 mm

#### Data collection

Bruker APEXII CCD diffractometer
Absorption correction: multi-scan (*SADABS*; Bruker, 2001 ▶) *T*_min_ = 0.939, *T*_max_ = 0.967
5239 measured reflections
1442 independent reflections
1269 reflections with *I* > 2σ(*I*)
*R*_int_ = 0.016

#### Refinement

*R*[*F* ^2^ > 2σ(*F* ^2^)] = 0.032
*wR*(*F* ^2^) = 0.099
*S* = 1.10
1442 reflections
104 parameters
H atoms treated by a mixture of independent and constrained refinement
Δρ_max_ = 0.25 e Å^−3^
Δρ_min_ = −0.29 e Å^−3^

### 

Data collection: *APEX2* (Bruker, 2007 ▶); cell refinement: *SAINT* (Bruker, 2007 ▶); data reduction: *SAINT*; program(s) used to solve structure: *SHELXS97* (Sheldrick, 2008 ▶); program(s) used to refine structure: *SHELXL97* (Sheldrick, 2008 ▶); molecular graphics: *PLATON* (Spek, 2009 ▶); software used to prepare material for publication: *PLATON*.

## Supplementary Material

Crystal structure: contains datablock(s) I, global. DOI: 10.1107/S1600536812024865/su2438sup1.cif

Structure factors: contains datablock(s) I. DOI: 10.1107/S1600536812024865/su2438Isup2.hkl

Supplementary material file. DOI: 10.1107/S1600536812024865/su2438Isup3.cml

Additional supplementary materials:  crystallographic information; 3D view; checkCIF report

## Figures and Tables

**Table 1:** Hydrogen-bond geometry (Å, °)

*D*—H⋯*A*	*D*—H	H⋯*A*	*D*⋯*A*	*D*—H⋯*A*
O1—H1*O*⋯O2^i^	0.83 (2)	2.09 (2)	2.9095 (12)	173 (2)
C3—H3⋯O3^ii^	0.95	2.54	3.3799 (14)	148

Symmetry codes: (i) 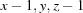; (ii) 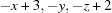.

**Fig. 1.**
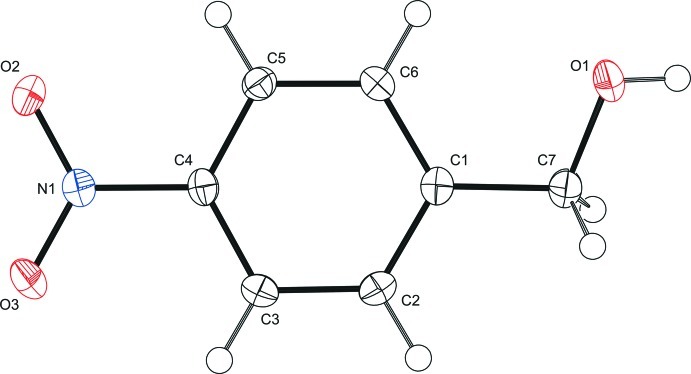
A view of the molecular structure of the title compound, showing the atom-labeling scheme. The displacement ellipsoids are drawn at the 50% probability level.

**Fig. 2.**
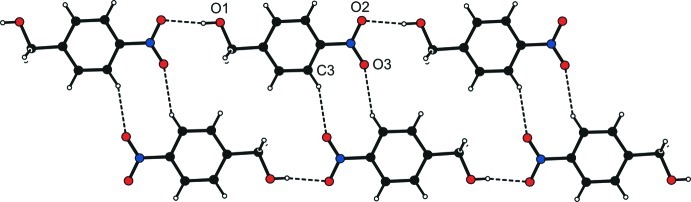
A section of H-bonded ribbons in the structure of the title compound. Hydrogen bonds are indicated with dashed lines.

**Fig. 3.**
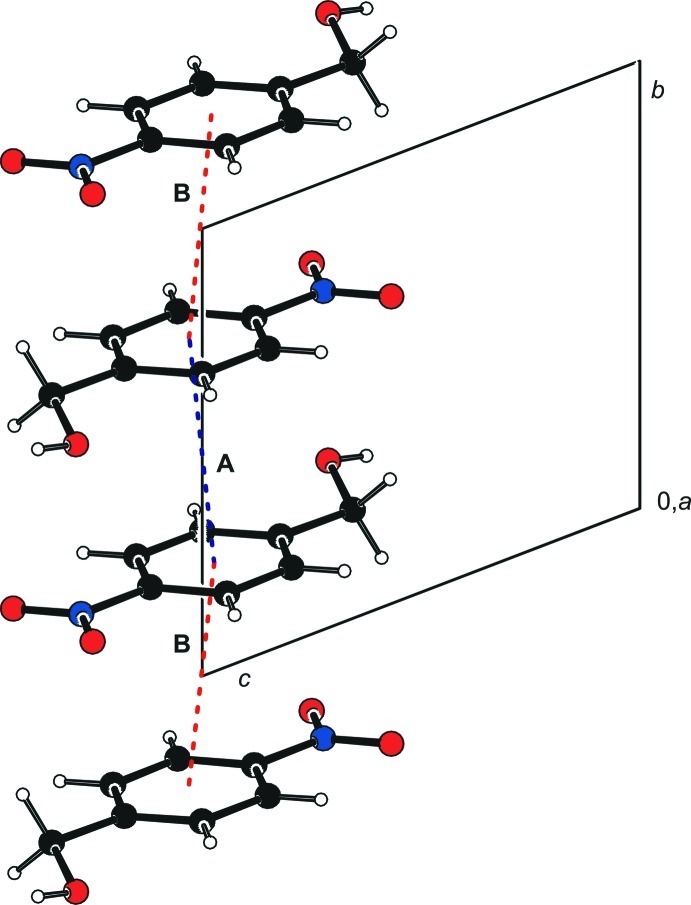
A view along the a axis of the π···π stacking interactions of individual molecules in the structure of the title compound.

**Fig. 4.**
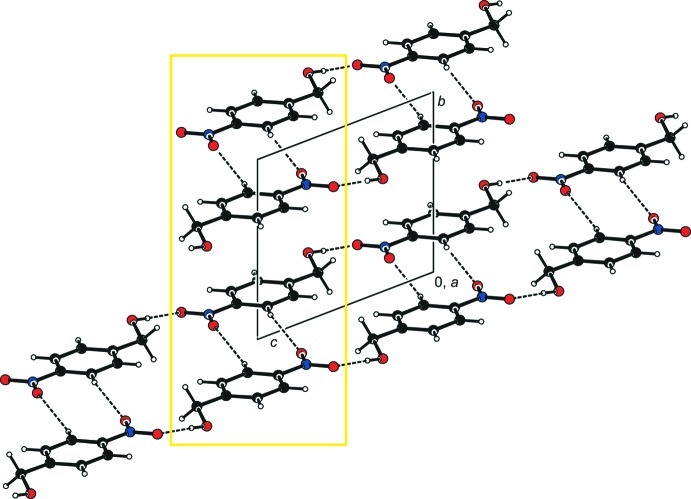
A view along the a axis of the crystal packing of the title compound, highlighting the interplay of hydrogen bonding (see Table 1) and π···π interactions [The yellow box limits the section presented in Fig. 3].

## supplementary crystallographic information

### Comment

The title compound, (4-nitrophenyl)methanol [common name: 4-nitrobenzyl alcohol], is a readily available reactive organic building block, commonly used in many organic reactions. As it can separate from the reaction mixture in the form of thin crystals and thus be mistakenly taken for the desired product (D. Drahoňovský, private communication), we decided to determined its molecular structure which surprisingly was unknown. The only structurally characterized compound comprising the title alcohol reported to date is a Zn(II) complex, [ZnL_2_(4-O_2_NC_6_H_4_CH_2_OH)_2_(H_2_O)_2_](NTf_2_)_2_ [where L = 1-(trifluoromethyl)-1,2-benziodoxol-3(1*H*)-one and Tf = CF_3_SO_3_], isolated from the reaction mixture after zinc-catalysed trifluoromethylation of alcohols with L and Zn(NTf_2_)_2_ (Koller *et al.*, 2009).

The molecular geometry of the title compound, Fig. 1, is rather unexceptional with bond distances falling in the usual ranges (Allen *et al.*, 2006). The substituents in the *para* positions of the benzene ring bind symmetrically as indicated by the O2/O3—N1—C4 angles of 118.10 (9) ° and 119.34 (9) ° for the NO_2_ group and by the angles C2/C6—C1—C7 of 119.21 (10) ° and 121.32 (10) ° for the CH_2_OH moiety. The nitro group is rotated from the plane of the benzene ring by as little as 0.44 (13) °. On the other hand, the hydroxy group is displaced from the plane of the central ring, being rotated by the pivotal C1—C7 bond. The perpendicular distance of O1 atom from the plane of the benzene ring is 0.356 (1) Å and the torsion angle C6—C1—C7—O1 is 16.73 (16) °. A relatively small but statistically significant difference observed for the individual N—O distances [viz: N1—O2 = 1.2369 (12) Å and N1—O3 = 1.2240 (13) Å] can be accounted for by different intermolecular interactions in which the respective NO_2_ oxygen atoms participate.

In the crystal, molecules associate into ribbons via a combination of O—H···O and C—H···O hydrogen bonds (Fig. 2 and Table 1). The shorter O1—H1O···O2 interactions result in the formation of infinite chains from molecules related by translation in the [1 0 1] direction, whereas the soft C3—H3···O3 contacts are formed between inversion-related molecules and thus cross-link the chains with their parallel, inversion-related counterparts into infinite ribbons oriented parallel to the *ac* plane.

Furthermore, the molecules associate into columnar stacks oriented along the crystallographic *b*-axis by means of π···π interactions (Figs. 3 and 4). The interacting molecules lie across crystallographic inversion centers and are therefore exactly parallel. Mutual offset of the interacting molecules by ca. 1.4 Å (pairs A in Fig. 3) and 1.7 Å (pairs B in Fig. 3) allows for efficient interactions between the the π-systems of the benzene rings and also for interactions between the π-systems of the benzene rings and the nitro groups. Distances of the centroids of the benzene rings are 3.6514 (7) Å for molecules located around inversion centres at b/2 (pairs A) and 3.8044 (7) Å for molecules related by the inversion centres at b = 0 and 1 (pairs B). Distances of the nitrogen atom from atom C1 in pairs A and B are ca. 4.2 Å and ca. 4.4 Å, respectively.

### Experimental

Yellowish prismatic crystals suitable for X-ray diffraction analysis were selected directly from a commercial sample of the title compound (Aldrich, 99%). Attempts to recrystallize the compound from hot heptane led only to very thin, plate-like crystal aggregates, which were not suitable for -ray diffraction analysis.

### Refinement

The OH hydrogen atom was located in a difference electron density map and refined freely. The C-bound H atoms were included in calculated positions and refined as riding atoms: C-H = 0.95 and 0.97 Å for aromatic and methylene H atoms, respectively, with *U*_iso_(H) =1.2*U*_eq_(C).

### Crystal data

**Table:**

C_7_H_7_NO_3_	*Z* = 2
*M**_r_* = 153.14	*F*(000) = 160
Triclinic, *P*1	*D*_x_ = 1.521 Mg m^−^^3^
Hall symbol: -P 1	Mo *K*α radiation, λ = 0.71073 Å
*a* = 6.2216 (5) Å	Cell parameters from 3056 reflections
*b* = 7.4096 (6) Å	θ = 2.9–27.0°
*c* = 7.7833 (6) Å	µ = 0.12 mm^−^^1^
α = 110.867 (2)°	*T* = 150 K
β = 93.667 (2)°	Prism, colorless
γ = 90.748 (3)°	0.53 × 0.31 × 0.28 mm
*V* = 334.34 (5) Å^3^	

### Data collection

**Table:**

Bruker APEXII CCD diffractometer	1442 independent reflections
Radiation source: fine-focus sealed tube	1269 reflections with *I* > 2σ(*I*)
Graphite monochromator	*R*_int_ = 0.016
φ and ω scans	θ_max_ = 27.0°, θ_min_ = 2.8°
Absorption correction: multi-scan (*SADABS*; Bruker, 2001)	*h* = −7→7
*T*_min_ = 0.939, *T*_max_ = 0.967	*k* = −9→9
5239 measured reflections	*l* = −9→9

### Refinement

**Table:**

Refinement on *F*^2^	Primary atom site location: structure-invariant direct methods
Least-squares matrix: full	Secondary atom site location: difference Fourier map
*R*[*F*^2^ > 2σ(*F*^2^)] = 0.032	Hydrogen site location: inferred from neighbouring sites
*wR*(*F*^2^) = 0.099	H atoms treated by a mixture of independent and constrained refinement
*S* = 1.10	*w* = 1/[σ^2^(*F*_o_^2^) + (0.0549*P*)^2^ + 0.0737*P*] where *P* = (*F*_o_^2^ + 2*F*_c_^2^)/3
1442 reflections	(Δ/σ)_max_ < 0.001
104 parameters	Δρ_max_ = 0.25 e Å^−^^3^
0 restraints	Δρ_min_ = −0.29 e Å^−^^3^

### Special details

**Table:**

Geometry. All e.s.d.'s (except the e.s.d. in the dihedral angle between two l.s. planes) are estimated using the full covariance matrix. The cell e.s.d.'s are taken into account individually in the estimation of e.s.d.'s in distances, angles and torsion angles; correlations between e.s.d.'s in cell parameters are only used when they are defined by crystal symmetry. An approximate (isotropic) treatment of cell e.s.d.'s is used for estimating e.s.d.'s involving l.s. planes.
Refinement. Refinement of *F*^2^ against all diffractions. The weighted *R*-factor *wR* and goodness of fit *S* are based on *F*^2^, conventional *R*-factors *R* are based on *F*, with *F* set to zero for negative *F*^2^. The threshold expression of *F*^2^ > σ(*F*^2^) is used only for calculating *R*-factors(gt) *etc*. and is not relevant to the choice of reflections for refinement. *R*-factors based on *F*^2^ are statistically about twice as large as those based on *F*, and *R*- factors based on all data will be even larger.

### Fractional atomic coordinates and isotropic or equivalent isotropic displacement parameters (Å^2^)

**Table:**

	*x*	*y*	*z*	*U*_iso_*/*U*_eq_	
O1	0.55157 (14)	0.37365 (14)	0.71166 (12)	0.0289 (2)	
H1O	0.459 (3)	0.352 (3)	0.625 (3)	0.059 (6)*	
O2	1.19725 (14)	0.31301 (14)	1.43192 (11)	0.0291 (2)	
O3	1.44094 (13)	0.17141 (12)	1.25026 (12)	0.0260 (2)	
N1	1.26402 (15)	0.24252 (13)	1.27641 (13)	0.0185 (2)	
C1	0.86002 (18)	0.24567 (15)	0.82183 (15)	0.0164 (2)	
C2	1.06259 (18)	0.16628 (15)	0.79623 (15)	0.0187 (3)	
H2	1.1093	0.1130	0.6751	0.022*	
C3	1.19706 (17)	0.16352 (15)	0.94381 (15)	0.0177 (3)	
H3	1.3348	0.1088	0.9260	0.021*	
C4	1.12356 (17)	0.24351 (14)	1.11879 (14)	0.0158 (2)	
C5	0.92362 (18)	0.32390 (15)	1.14985 (15)	0.0173 (2)	
H5	0.8781	0.3776	1.2714	0.021*	
C6	0.79120 (17)	0.32436 (15)	0.99981 (15)	0.0172 (2)	
H6	0.6531	0.3784	1.0182	0.021*	
C7	0.71876 (19)	0.24303 (16)	0.65630 (15)	0.0215 (3)	
H7A	0.8064	0.2792	0.5710	0.026*	
H7B	0.6562	0.1109	0.5901	0.026*	

### Atomic displacement parameters (Å^2^)

**Table:**

	*U*^11^	*U*^22^	*U*^33^	*U*^12^	*U*^13^	*U*^23^
O1	0.0235 (5)	0.0379 (5)	0.0213 (4)	0.0103 (4)	−0.0055 (3)	0.0068 (4)
O2	0.0257 (5)	0.0455 (6)	0.0151 (4)	0.0062 (4)	0.0000 (3)	0.0098 (4)
O3	0.0197 (4)	0.0303 (5)	0.0278 (5)	0.0086 (3)	−0.0016 (3)	0.0103 (4)
N1	0.0176 (5)	0.0194 (5)	0.0191 (5)	0.0006 (3)	−0.0008 (4)	0.0081 (4)
C1	0.0184 (5)	0.0137 (5)	0.0171 (5)	−0.0021 (4)	−0.0014 (4)	0.0060 (4)
C2	0.0217 (6)	0.0175 (5)	0.0155 (5)	0.0010 (4)	0.0034 (4)	0.0041 (4)
C3	0.0160 (5)	0.0162 (5)	0.0209 (6)	0.0024 (4)	0.0028 (4)	0.0062 (4)
C4	0.0161 (5)	0.0145 (5)	0.0172 (5)	−0.0011 (4)	−0.0020 (4)	0.0066 (4)
C5	0.0180 (5)	0.0185 (5)	0.0154 (5)	0.0007 (4)	0.0022 (4)	0.0058 (4)
C6	0.0146 (5)	0.0171 (5)	0.0201 (5)	0.0015 (4)	0.0010 (4)	0.0071 (4)
C7	0.0225 (6)	0.0235 (6)	0.0173 (5)	0.0031 (4)	−0.0015 (4)	0.0063 (4)

### Geometric parameters (Å, º)

**Table:**

O1—C7	1.4110 (13)	C2—H2	0.9500
O1—H1O	0.82 (2)	C3—C4	1.3881 (15)
O2—N1	1.2369 (12)	C3—H3	0.9500
O3—N1	1.2239 (12)	C4—C5	1.3842 (15)
N1—C4	1.4623 (13)	C5—C6	1.3865 (15)
C1—C2	1.3936 (15)	C5—H5	0.9500
C1—C6	1.3961 (15)	C6—H6	0.9500
C1—C7	1.5065 (14)	C7—H7A	0.9900
C2—C3	1.3835 (15)	C7—H7B	0.9900
			
C7—O1—H1O	109.2 (14)	C5—C4—N1	118.87 (10)
O3—N1—O2	122.56 (9)	C3—C4—N1	118.37 (10)
O3—N1—C4	119.34 (9)	C4—C5—C6	118.59 (10)
O2—N1—C4	118.10 (9)	C4—C5—H5	120.7
C2—C1—C6	119.46 (10)	C6—C5—H5	120.7
C2—C1—C7	119.21 (10)	C5—C6—C1	120.24 (10)
C6—C1—C7	121.32 (10)	C5—C6—H6	119.9
C3—C2—C1	121.30 (10)	C1—C6—H6	119.9
C3—C2—H2	119.3	O1—C7—C1	110.28 (9)
C1—C2—H2	119.3	O1—C7—H7A	109.6
C2—C3—C4	117.65 (10)	C1—C7—H7A	109.6
C2—C3—H3	121.2	O1—C7—H7B	109.6
C4—C3—H3	121.2	C1—C7—H7B	109.6
C5—C4—C3	122.76 (10)	H7A—C7—H7B	108.1

### Hydrogen-bond geometry (Å, º)

**Table:**

*D*—H···*A*	*D*—H	H···*A*	*D*···*A*	*D*—H···*A*
O1—H1*O*···O2^i^	0.83 (2)	2.09 (2)	2.9095 (12)	173 (2)
C3—H3···O3^ii^	0.95	2.54	3.3799 (14)	148

Symmetry codes: (i) *x*−1, *y*, *z*−1; (ii) −*x*+3, −*y*, −*z*+2.
